# Predicting Preterm Labour: Current Status and Future Prospects

**DOI:** 10.1155/2015/435014

**Published:** 2015-06-15

**Authors:** Harry M. Georgiou, Megan K. W. Di Quinzio, Michael Permezel, Shaun P. Brennecke

**Affiliations:** ^1^Department of Obstetrics and Gynaecology, University of Melbourne, VIC 3010, Australia; ^2^Mercy Perinatal Research Centre, Mercy Hospital for Women, Heidelberg, VIC 3084, Australia; ^3^Pregnancy Research Centre, Royal Women's Hospital, Parkville, VIC 3052, Australia

## Abstract

Preterm labour and birth are a major cause of perinatal morbidity and mortality. Despite modern advances in obstetric and neonatal management, the rate of preterm birth in the developed world is increasing. Yet even though numerous risk factors associated with preterm birth have been identified, the ability to accurately predict when labour will occur remains elusive, whether it is at a term or preterm gestation. In the latter case, this is likely due to the multifactorial aetiology of preterm labour wherein women may display different clinical presentations that lead to preterm birth. The discovery of novel biomarkers that could reliably identify women who will subsequently deliver preterm may allow for timely medical intervention and targeted therapeutic treatments aimed at improving maternal and fetal outcomes. Various body fluids including amniotic fluid, urine, saliva, blood (serum/plasma), and cervicovaginal fluid all provide a rich protein source of putative biochemical markers that may be causative or reflective of the various pathophysiological disorders of pregnancy, including preterm labour. This short review will highlight recent advances in the field of biomarker discovery and the utility of single and multiple biomarkers for the prediction of preterm birth in the absence of intra-amniotic infection.

## 1. The Problem of Preterm Birth

Preterm birth (<37 weeks' gestation) is the most significant clinical problem facing contemporary obstetrics in the developed world. Preterm birth occurs in 5–18 percent of all deliveries worldwide with most developed countries reporting an increased incidence over the last 3 decades [1]. It is estimated that 15 million preterm births occur each year with 1.1 million infants dying from preterm birth complications. Fifteen populous countries (including the USA) account for 75 percent of these deaths [2]. The significance of premature birth cannot be underestimated. Being born too early is the major cause of perinatal morbidity and mortality accounting for 85 percent of all early infant deaths, not secondary to congenital abnormality [3]. Advances in perinatology and neonatology in the past decade have resulted in increased survival rates, particularly for the extremely premature baby (born between 24 and 27 weeks' gestation) but unfortunately the associated morbidity for these survivors remains significant where one-fifth to one-quarter will suffer at least one major disability including chronic lung disease, impaired mental development, cerebral palsy, deafness, or blindness [4, 5]. Even late preterm infants (born between 32 and 36 weeks' gestation) have a greater risk of respiratory distress syndrome, feeding difficulties, temperature instability, jaundice, and delayed brain development [6].

Aside from the medical implications of preterm labour and delivery, there is a considerable fiscal challenge to society in terms of providing appropriate short- and long-term medical care. Data from the USA indicate that hospital care of a premature infant is, on average, 10 times higher than an infant delivered at term. In 2005, the preterm birth burden in the USA was $26.2 billion in medical and educational and lost productivity costs [1, 2]. Another cost, which is difficult to measure, is the emotional and psychological impact on these babies and their families.

## 2. Causes of Preterm Birth

Premature birth may be iatrogenic or spontaneous. Iatrogenic premature birth is the result of a medical intervention due to a fetal and/or maternal condition (e.g., fetal growth restriction, preeclampsia) necessitating early delivery. By contrast, spontaneous premature birth often occurs despite best efforts to prolong the pregnancy. It is estimated that up to 80 percent of premature births fall into this category. The major goal of the obstetrician in this regard is therefore to prevent preterm birth. Failing in this, it is crucial to delay preterm birth long enough to optimise the outcome for the fetus, for example, to allow for the transfer of the pregnant woman to a healthcare centre with appropriate neonatal facilities, to administer corticosteroids to enhance fetal lung maturation, and/or to give magnesium for fetal neuroprotection. A prerequisite for the success of this strategy is the reliable prediction/identification of women at risk of preterm birth.

Evidence suggests that spontaneous preterm labour and delivery are a heterogeneous condition with many triggers or precipitating factors including maternal genital tract haemorrhage, cervical dysfunction, idiopathic uterine contractions, infection, malnutrition, multifetal pregnancy, and spontaneous rupture of the fetal membranes [7]. Four distinct mechanisms for the pathogenesis of preterm labour have been described and include premature activation of the fetal hypothalamic pituitary axis, mechanical stretch, inflammation/matrix remodelling, and placental abruption [8]. The temporal convergence of cervical effacement and dilatation, myometrial activation, and the rupture of fetal membranes are common to all spontaneous labour and in all placental mammals irrespective of the initiating trigger(s) or whether labour occurs at a term or at preterm gestation.

## 3. Rationale behind Screening for and Managing Preterm Labour

While our understanding of human labour and the causes of preterm labour have advanced over the past decades, the ability to accurately predict when preterm labour or preterm prelabour rupture of membranes (PROM) will occur has remained elusive. As a consequence the development of targeted preventative therapies directed at specific at-risk subpopulations has been impeded.

The current management of women deemed to be at risk of preterm birth depends upon clinical presentation. Apart from modifying lifestyle,* asymptomatic* women with known risk factors (see below) may benefit from progesterone supplementation (usually administered as a daily vaginal pessary), as several systematic reviews of randomised trials have demonstrated a reduced incidence of preterm birth in at-risk women [9–11]. In women presenting with* symptoms* of preterm labour (“threatened” preterm labour), our understanding of the pathophysiology has facilitated the development of safer and more selective therapies used to suppress uterine activity (tocolytics) and include calcium channel blockers, nitric oxide donors, prostaglandin synthase inhibitors, cyclooxygenase inhibitors, and oxytocin receptor antagonists [12, 13], while antibiotics (to treat infection), corticosteroids (to aid fetal lung development), and magnesium sulphate (serving as a neuroprotectant) also have a place as prophylactic therapies.

Recognition of the subgroups of women who may benefit from these or other therapeutic approaches would be optimal. This, however, relies upon (i) a better understanding of the mechanism(s) of labour and the causes of spontaneous preterm labour, (ii) the identification of biomarker(s) for the early and reliable prediction of spontaneous preterm labour, (iii) the allocation of at-risk individuals to appropriate models of antenatal care and clinical surveillance, (iv) the early commencement of prophylactic therapies, and (v) recruitment of high-risk individuals to clinical trials for the development of optimal therapies to mitigate specific adverse outcomes [14].

## 4. Current Approaches to the Prediction of Preterm Labour

Current screening tests for the prediction of spontaneous preterm labour can be divided into three general categories: (i) risk factor assessment, (ii) cervical measurement, and (iii) biochemical markers; however, it should be emphasised that significant associations with labour may not necessarily translate into clinical predictive utility.

### 4.1. Risk Factor Assessment

Clinical risk factors for preterm birth include (i) demographic characteristics such as low socioeconomic status, poor antenatal care, extremes of maternal age, or malnutrition, (ii) behavioural factors including smoking, illicit drug use, alcohol consumption, or heavy physical work, (iii) obstetric history including familial (genetic) predisposition, uterine malformation, previous preterm labour or preterm PROM, previous cone biopsy or cervical surgery, and (iv) aspects of the current pregnancy such as multifetal gestation, genital tract bleeding and/or infection, fetal malformation, preterm rupture of membranes, shortened cervix, and other pregnancy complications including preeclampsia and gestational diabetes mellitus [15, 16]. A previous preterm birth before 34 weeks' gestation is amongst the strongest risk factors for subsequent preterm birth with a relative risk of 13.56 [17]. However, insofar as nulliparous women have no past obstetric history to call upon, any such previous history risk factor-based assessment is inapplicable in their situation. Overall risk factor assessment alone is unreliable, as over 50 percent of pregnancies that deliver preterm will fail to be identified [3, 15, 18].

### 4.2. Cervical Measurement

In some women, a shortened cervical length can be due to natural biological variation. In other cases early cervical shortening or effacement may be due to haemorrhage or infection leading to inflammation, or due to biophysical effects of uterine overdistension (e.g., multifetal gestation) or subclinical contractions. Using transvaginal ultrasound, a cervical length below the 10th centile for gestational age increased by 6-fold the risk of delivery prior to 35 weeks' gestation [3]. A review of 35 studies using sonographically assessed cervical length to predict preterm delivery in* asymptomatic* women and found sensitivities ranging from 68% to 100% and specificities from 44% to 79% with wide variations in their predictive values [19]. A more recent meta-analysis of 28 studies assessing cervical length (<15 mm) in* symptomatic* women with threatened preterm labour found sensitivities ranging from 53% to 67% and specificities ranging from 89% to 92% for delivery within one week [20]. Due to limitations in ultrasound availability and operator expertise, cervical length alone cannot be reliably utilised to predict preterm labour or used as a routine screening tool [21]. Nevertheless, with the exception of modifying lifestyle and/or treating known infection, cervical length determination possibly provides the best avenue for therapeutic intervention, at least at this time in the developed world.

### 4.3. Biochemical Markers

While the direct study of gestational tissues (e.g., vaginal epithelium, cervix, endometrium, myometrium, placenta, choriodecidua, and fetal membranes) may provide more accurate localised information on the state of a pregnancy and impending labour, it is the more easily accessible biological fluids including whole blood/serum/plasma, urine, saliva, amniotic fluid, and cervicovaginal fluid (CVF) that are more likely to be amenable to the creation of a rapid bedside biomarker test for predicting preterm labour or preterm PROM. These body fluids provide rich sources of proteins and metabolites that vary in concentration in response to pregnancy and adverse pregnancy states [22–25]. With the development of genomic and proteomic technologies over the past two decades the simultaneous screening of thousands of genes and gene products from small samples of tissue or body fluid has become possible [26–29]. However, it is becoming evident that single biomarker approaches for the early detection of preterm birth (in the absence of infection) may never achieve the desired diagnostic efficiency [23, 24, 30, 31].

#### 4.3.1. Amniotic Fluid

While the genome and proteome of amniotic fluid have been extensively investigated, particularly in the context of fetal chromosomal abnormality or infection (with or without clinical chorioamnionitis), the sampling of amniotic fluid (amniocentesis) is not likely to become routine practice solely for the purpose of preterm labour prediction. Indeed, the procedure* per se* can precipitate preterm labour as well as potentially causing fetal trauma and infection. In the absence of intra-amniotic infection, several protein biomarkers in human amniotic fluid including interleukin-6 (IL-6, symbols in parenthesis assigned by the HUGO Gene Nomenclature Committee) [32, 33], interleukin-8 (IL8) [34], interleukin-16 (IL16) [35], interferon gamma-inducible protein 10 (CXCL10) [33], annexin A2 (ANXA2) [34], and other proinflammatory proteins (CXCL11, ADAM8, SLPI, sICAM1, and vICAM1) [36] have been found to be associated with increased incidence of preterm labour or preterm PROM, yet other studies failed to confirm some of these findings [37]. Where predictive modelling has been performed, no biomarker in isolation appears to provide adequate predictive efficacy, with generally poor sensitivity and/or specificity.

#### 4.3.2. Saliva

Salivary progesterone has been investigated as a biomarker of preterm birth. A low saliva concentration of progesterone, obtained between 24 and 34 weeks of gestation, has been described in women at risk of early preterm labour (<34 weeks of gestation) [38]. This study was conducted on women with a singleton pregnancy with at least one risk factor for preterm birth. Fetal fibronectin (fFN, see below) was also measured at 24 and 27 weeks of gestation in the same cohort of women. However, no observed correlation between fFN and salivary progesterone was demonstrated. A subsequent study performed by Priya et al. [39] examined the predictive utility of salivary progesterone for preterm birth. This study also included women with a singleton pregnancy and at least one identifiable risk factor for preterm birth. Salivary progesterone was measured at 24 to 28 weeks of gestation and repeated after 3-4 weeks. A single cut-off value for salivary progesterone of 2575 pg/mL produced a sensitivity of 83%, specificity 86%, positive predictive value 60%, and negative predictive value 95%, identifying more than 80% of women who delivered before 34 weeks of gestation. The authors propose that estimating salivary progesterone in high-risk pregnant women may identify those in whom benefit may be derived from supplemental progesterone therapy.

#### 4.3.3. Urine

There is a paucity of data examining chemical biomarkers of preterm birth in urine. With the exception of screening pregnant women for asymptomatic bacteraemia, where antibiotic treatment reduces the risk of infection-mediated preterm birth, little is known of the specific inflammatory mediators that may trigger spontaneous preterm labour. Hanna and Kiefer [40] performed a pilot study of 20 women undergoing elective lower uterine segment Caesarean section at term. Maternal urine, blood, saliva, vaginal and cervical secretions, amniotic fluid, and placental samples were obtained and directly compared in order to elucidate the inflammatory/immunological mediators associated with each compartment. There was very little overlap between each compartment, with the exception being a significant correlation between vaginal and cervical samples.

#### 4.3.4. Blood (Serum or Plasma)

While blood is easily accessible, allowing for rapid sampling that is minimally invasive, its relatively large volume and remote proximity from gestational tissues suggest that chemical biomarkers associated with impending labour may be diluted amongst the thousands of other serum/plasma proteins. The fact that many proteins derived from gestational tissues also reside in the peripheral circulation may further skew any meaningful interpretation of their abundance in relation to labour.

Despite these logistical challenges investigators continue to search for blood-borne biomarkers that may be useful predictors of labour.

There is a large body of literature, including several reviews [22, 24, 41–43] assessing numerous blood-borne biochemical markers for the prediction of spontaneous preterm labour. Although numerous case-control studies reveal significant differences in a number of biochemical markers, few of these provide adequate predictive efficacy [44]. A promising study of plasma urocortin concentration in women with symptoms of threatened preterm labour displayed a sensitivity of 80%, a specificity of 100% with a positive predictive value of 100% for preterm delivery within 7 days of sampling [45]. It should be emphasized, however, that these women were essentially in labour as all experienced painful uterine contractions in less than 5 min intervals had dilatation or effacement of the cervix and/or ruptured membranes. There appears to be no follow-up to this study. Using multiplex analyte profiling (xMAP) technology, Tsiartas et al. [46] measured 27 proteins in women presenting with threatened preterm labour. While several proteins were significantly differentially expressed (interleukin-10 (IL-10), soluble interleukin-6 receptor alpha (sIL6R), tumour necrosis factor-beta (LTA), macrophage inflammatory protein-1 alpha (CCL4), matrix metalloproteinase-9 (MMP9), brain-derived neurotrophic factor (BDNF), granulocyte-monocyte-colony-stimulating factor (CSF2), and soluble tumour necrosis factor receptor I (sTNFR1A)), the measurement of cervical length alone provided a greater predictive odds ratio than any of the single biochemical markers studied.

#### 4.3.5. Cervicovaginal Fluid

Interest in the human CVF as a potential diagnostic tool is highlighted by the rapid succession of publications over the past decade describing the nonpregnant and pregnant CVF proteome [47–54]. The CVF is a complex mixture of secretions derived from the vagina, endocervix, endometrial decidua, and amniochorion and therefore serves as an important diagnostic site to monitor maternal and fetal health in pregnancy. Unlike the amniotic fluid the CVF is readily accessible and collection is minimally invasive and safe. There are two commonly used clinical biomarker tests for the prediction of preterm labour, namely, fetal fibronectin (fFN) and phosphorylated insulin-like growth factor binding protein-1 (phIGFBP1).

fFN is a large molecular weight glycoprotein produced by the trophoblast that serves to maintain the chorionic-decidual extracellular matrix. Beyond 16–20 weeks' gestation fFN is not detectible in the CVF. If found beyond 20 weeks' gestation, it may suggest a disruption of the choriodecidual interface and has been identified as a predictor of spontaneous preterm labour [55]. A meta-analysis examining the utility of fFN to predict preterm birth within 7–14 days in* symptomatic* women reported 78–89% sensitivity and 86% specificity [56]. In the same review the utility of fFN testing in* asymptomatic* women found a lower sensitivity (68–76%) but comparable specificity (88-89%) in predicting spontaneous preterm birth within the 7–14 days. While the fFN test appears to be more informative in women presenting with threatened preterm labour [57, 58], due to its generally poor positive predictive value [59] and limitations due to external factors (e.g., amniotic fluid contamination, vaginal bleeding, and unprotected sexual intercourse), the fFN test has limited application. fFN is used clinically for its negative predictive value, which exceeds 95% in some studies [56] and is particularly useful in determining whether a symptomatic patient in a remote region requires emergency transfer to a tertiary healthcare facility.

phIGFBP1 is secreted by decidual cells and leaks into cervical secretions when fetal membranes detach from decidua. It has been used to clinically assess cervical maturation. Clinical diagnostic trials indicate that, like fFN, phIGFBP1 is a good negative predictor of preterm birth (92% specificity) but lacks suitable sensitivity and positive predictive value in* asymptomatic* women [60, 61]. Clearly there is a need for improved biomarker predictive test(s) for preterm labour than currently available tests.

Numerous studies have explored a myriad number of biochemical markers in the human CVF for the prediction of preterm birth. While a thorough description of all these studies is beyond the scope of this review, several other excellent reviews have been published over the past decade describing numerous biochemical markers associated with preterm labour [24, 31, 41, 42, 62, 63]. However, only relatively few studies have analysed the predictive efficacy of these biomarkers and careful consideration is required to distinguish pregnancies experiencing spontaneous preterm labour, spontaneous preterm PROM, and symptomatic (threatened) preterm labour (in the absence of infection). A common thread amongst several studies is IL6 [41, 42, 64, 65]. Taylor et al. [65] examined vaginal fluid for various inflammatory makers and reported increased IL6 in asymptomatic women with subsequent spontaneous preterm delivery. IL6 was able to predict preterm delivery in these women with 43% sensitivity and 74% specificity. As noted by Menon et al. [42], any consideration of the currently available published data is hard-pressed “to identify a single biomarker that stands out in its ability to predict spontaneous preterm birth….”

Using 2D electrophoresis, di Quinzio et al. were the first to publish a “2D map” of the human CVF proteome in pregnancy [47]. More extensive CVF proteome maps were subsequently published by the same group [51, 54]. Using various sophisticated proteomic technologies over the past decade, researchers have gained considerable insight into the complex array of proteins present in the human CVF (well over 600 different peptides have now been described) [48–54]. However, until recently, only a very small number of proteomic studies have investigated changes in protein expression in association with human labour [66, 67].

In early 2D electrophoresis biomarker-discovery studies [51, 54], differentially expressed proteins that preceded spontaneous* term* labour were identified and characterised by mass spectrometry and included several that were significantly downregulated: protease inhibitors including cystatin A (CSTA), monocyte/neutrophil elastase inhibitor (SERPINB1), squamous cell carcinoma antigen 1 (SERPINB3), and squamous cell carcinoma antigen 2 (SERPINB4); an anti-inflammatory cytokine, interleukin-1 receptor antagonist (IL1RN); a number of antioxidant enzymes including thioredoxin-1 (TXN), Cu,Zn-superoxide dismutase (SOD1), peroxiredoxin-2 (PRDX2), and glutathione S-transferase pi (GSTP1), while others were significantly upregulated including inhibitors of phospholipid metabolism, epidermal fatty acid binding protein 5 (FABP5), and annexin A3 (ANXA3) as well as albumin (ALB). Indeed, a later study confirmed that the total antioxidant potential of CVF is significantly reduced with impending* term* labour [68]. These findings provide potentially unique informative insights into possible mechanisms involved in the onset of labour and offer promising options for biomarker development.

Although informative, discovery based proteomic methods are only semiquantitative, and therefore further quantitative studies to determine accurate protein expression are required for biomarker development. To this end, candidate CVF biomarkers have been evaluated [68–72] in women who experienced spontaneous labour between 37 and 41 weeks' gestation (in some cases up to 300 CVF samples), using traditional immune-based or enzyme activity methods. Preliminary predictive modelling was performed and a number of interesting findings arose from these analyses.

*Interleukin-1 Family.* In a series of studies, IL1RN was investigated together with the proinflammatory cytokines interleukin-1 alpha and interleukin-1 beta (IL1A and IL1B). These cytokines all compete for the same interleukin-1 receptor. As expected, while IL1A and IL1B concentrations peaked 4–14 d before labour, the anti-inflammatory cytokine IL1RN significantly decreased with impending spontaneous labour [69, 70].

*Antioxidants.* Three measures of oxidative stress status were determined. TXN was significantly reduced in-labour while SOD1 showed a significant linear decrease with impending labour. These findings prompted an assessment of the total antioxidant capacity (TAC) of CVF where it was found to significantly decrease with approaching labour. These findings confirmed that not only is labour associated with increased oxidative stress and lowered antioxidant capacity, but also these changes precede labour onset [68].

*Proteases.* A number of cysteine proteases, cathepsins B, H, L, and S (CTSB, CTSH, CTSL, and CTSS) and their inhibitor, CSTA, were measured in the CVF at* term* pregnancy. While the proteases were either undetected or unaltered, CSTA was confirmed to significantly decrease with approaching labour [71]. Using xMAP technology, a panel of matrix metalloproteinases (MMP1, 2, 3, 7, 8, 9, 12, and 13) and the tissue inhibitors of metalloproteinases (TIMP1 and TIMP2) were simultaneously investigated. Not only were MMP7 and TIMP2 found to be significantly elevated during labour, but TIMP1 was also significantly elevated up to eight days before the onset of labour [72]. This was the first comprehensive study of MMPs and TIMPs in the human CVF and the findings demonstrate a delicate regulation of proteases and their inhibitors during matrix remodelling and cervical ripening.

More recently, the same group has completed a series of studies in the* preterm* pregnancy setting and evaluated a number of biomarkers previously identified in the* term* pregnancy studies [73–76]. The premise of these studies was that a common final pathway of labour exists, irrespective of the initiating trigger(s), comprising cervical effacement and dilatation, myometrial activation, and the rupture of fetal membranes. Two cohorts were investigated: (i)* asymptomatic* pregnant women with known risk factors of preterm labour [73, 74] or preterm PROM [75] and (ii) women with* symptoms* of “threatened” preterm labour [76].

*Spontaneous Preterm Labour in At-Risk Women.* Using 2D gel electrophoresis techniques (2D-DIGE and 2D-PAGE), the CVF proteome of women who spontaneously delivered preterm (11 to 22 days prior to labour onset) was compared with gestation-matched women who delivered at term. Five candidate biomarkers were selected for validation in a large independent cohort of* asymptomatic* women. TXN and IL1RN concentrations in the CVF were found to be significantly reduced up to 90 days prior to spontaneous preterm labour compared to gestation matched women who subsequently delivered at term. TXN was able to predict spontaneous preterm labour up to 28 days from labour with a high positive predictive value and negative predictive value of 75.0% and 96.4%, respectively. IL1RN also showed comparable positive and negative predictive values of 72.7% and 95.7%, respectively [73]. In a subsequent investigation, Vitamin D binding protein (GC, group-specific component) was measured throughout pregnancy. Compared to gestation-matched controls, women destined for a preterm labour had significantly elevated levels of GC up to 100 days before spontaneous labour. GC concentrations were significantly increased by up to 7-fold, 14 days before labour onset. Predictive modelling indicated that GC had a positive predictive value of 82.8% at 3 days and 78.8% at 7 days before labour onset [74]. In a study investigating spontaneous preterm PROM, both ILRN and CSTA in the CVF were found to be significantly reduced 6–23 days before membrane rupture [75].

*Threatened Preterm Labour.* Once again, using 2D gel electrophoresis techniques (2D-DIGE and 2D-PAGE), the CVF proteome of* symptomatic* women (but with no observable cervical change) who spontaneously delivered preterm within 7 days was compared with gestation-matched women who delivered at term. Four biomarkers, TXN, IL1RN, GC, and ALB were identified and further investigated and all four were significantly altered. From these studies optimal concentration thresholds were determined and predictive modelling was performed. GC displayed 77.8% sensitivity and 98.1% specificity while ALB displayed 83.3% sensitivity and 73.3% specificity [76].

An important consideration in all of these* term* and* preterm* pregnancy studies was to test the influence of potential confounder variables. Findings indicate that colonisation with common vaginal microflora (e.g.,* Ureaplasma* spp., Group B* Streptococci* spp., and Candida) have no effect on the expression of these biomarkers nor did multifetal gestation (twin pregnancy). However, it should be emphasised that women with vaginal bleeding, ruptured fetal membranes, or who had had unprotected sexual intercourse in the preceding 24 hrs or transvaginal ultrasound in the preceding 6 hours were excluded from these studies [68–71, 73, 74, 76].

These investigations indicate that although the “triggers” of labour onset may vary, the terminal mechanisms involved in both* term* and* preterm* labour and parturition are common, namely, matrix remodelling, fetal membranes rupture, and uterine contractions. These studies have also provided unique insights into the multiple mechanisms that culminate in labour and include inflammation (IL1B, IL1RN); matrix remodelling (CSTA, SERPINB1, SERPINB3, and SERPINB4), oxidative stress (TXN, SOD1, PRDX2, and GSTP1), and lipid metabolism (FABP5, ANXA3). It is for this reason that multiple biomarkers that target different pathways are likely to prove most beneficial in predicting spontaneous preterm labour, preterm PROM, and threatened preterm labour.

## 5. Future Approaches to the Prediction of Preterm labour

Identification of a single biomarker to predict spontaneous preterm labour poses a significant challenge due to the heterogeneity of clinical presentations and of the biochemical mechanisms involved in preterm birth (see Menon et al., guidelines for future biomarker studies [42]). Presently, none of the common late-pregnancy complications including preterm labour can be predicted with sufficient accuracy (sensitivity and specificity) using a single biochemical marker [22–24, 31, 42]. For this reason the simultaneous quantification of multiple biomarkers, that may include demographic/risk-factor(s), cervical length and biochemical marker(s), and the development of multivariate classification models represent a promising approach to improving diagnostic efficiency.

The ability to predict* term* labour within 3 days of onset utilising the interleukin-1 family of cytokines (IL1A, IL1B, and IL1RN) was assessed by Heng et al. [70]. While the sensitivity for the individual biomarkers was 52.8% with positive predictive values ranging from 25 to 33%, the combined model of all three cytokines displayed an area under curve of 0.963, with sensitivity of 86.1%, specificity of 91.7%, and positive and negative predictive values of 68.8% and 96.9%, respectively [70]. A similar study of* term* labour prediction from the same group demonstrated that two combined biomarkers of oxidative stress, SOD1 and total oxidant capacity, displayed superior predictive efficiency compared to either biomarker alone [68].

This group further demonstrated the advantage of multiple biomarker modelling using data derived from symptomatic women experiencing* preterm* labour. The combination of ALB and GC could predict labour up to 7 days prior to onset with a specificity of 100% and positive predictive value of 100%. By contrast, in the same cohort of women, fFN displayed a specificity of 85.3% and positive predictive value of only 30.8% [76], which is comparable to other published data.

Several other studies have reported increased odds ratios and/or predictive efficiency for preterm birth when two or more biomarkers (sometimes derived from different tissues) are combined compared to single biomarkers alone [15, 22, 43, 46, 62, 77, 78]. With advances in genomic, proteomic, and metabolomic technologies and the simultaneous screening of thousands of potential biomarkers, these studies highlight the advantage of multivariate regression modelling and an increased need for bioinformatic analysis due to the exponential growth in data acquisition.

## 6. Conclusion

The ability to accurately predict and therefore prevent preterm labour and birth remains one of the crucial challenges facing modern obstetrics. Identifying women who are most at risk of preterm birth would allow the tailoring of medical interventions and targeted therapeutic treatments aimed at improving maternal and fetal outcomes. Current predictive tests display poor positive predictive values and it is likely that no single biomarker will ever achieve the desired predictive efficacy due to the multifaceted aetiology of preterm birth. Therefore multiple biomarker modelling is receiving increased attention. To this end, the CVF is an ideal biological fluid for the discovery of molecular biomarkers associated with labour due to its proximity to the gestational tissues that undergo change with advancing gestation. Genomic, proteomic, and metabolomic approaches will ultimately enable the discovery of novel molecular biomarkers involved in the physiology of labour and pathophysiology of preterm birth, but it is becoming increasingly evident that different groups of biomarkers (perhaps comprising risk factors, cervical length, and molecular markers) may be required to distinguish pregnancies experiencing spontaneous preterm labour, spontaneous preterm PROM, and symptomatic (threatened) preterm labour, whether these are in the presence or absence of genital tract infection.
